# Gender differences in SCRABBLE performance and associated engagement in purposeful practice activities

**DOI:** 10.1007/s00426-017-0905-3

**Published:** 2017-08-31

**Authors:** Jerad H. Moxley, K. Anders Ericsson, Michael Tuffiash

**Affiliations:** 1 University of Miami Miller School of Medicine, Miami, USA; 2 Florida State University, Tallahassee, USA; 3 Center for Aging, Jackson Behavioral Health, 1695 NW 9th Ave, Miami, FL 33136 USA

## Abstract

In two studies, the SCRABBLE skill of male and female participants at the National SCRABBLE Championship was analyzed and revealed superior performance for males. By collecting increasingly detailed information about the participants’ engagement in practice-related activities, we found that over half of the variance in SCRABBLE performance was accounted for by measures of starting ages and the amount of different types of practice activities. Males and females did not differ significantly in the benefits to their performance derived from engagement in SCRABBLE-specific practice alone (purposeful practice). However, gender differences in performance were fully mediated by lower engagement in purposeful practice by females and by their rated preference for playing games of SCRABBLE—an activity where more extended engagement is not associated with increased SCRABBLE performance. General implications from our account of gender differences in skill acquisition are discussed, and future research is proposed for how the duration of engagement in effective deliberate practice can be experimentally manipulated.

**Electronic supplementary material:**

The online version of this article (doi:10.1007/s00426-017-0905-3) contains supplementary material, which is available to authorized users.

Gender gaps in academia, particularly in STEM fields, are currently of great national concern. In 2014, the then President, Barack Obama, framed gender differences as a matter of national economic interest, stating:

“There’s so much talent to be tapped if we’re working together and lifting it up. Right now, fewer than one in five bachelor’s degrees in engineering or computer science are earned by women. Fewer than three in ten workers in science and engineering are women. That means we’ve got half the field – or half our team we’re not even putting on the field. We’ve got to change those numbers. These are the fields of the future. This is where the good jobs are going to be. And I want America to be home for those jobs.” (White House, 2014)

President Obama assumes that men and women have equal or, at least similar, abilities to succeed in these fields and that lower participation rates by women imply that the United States is not maximizing its potential. Despite Obama’s influential view, the cause of differences in professional success between men and women is hotly debated in the social sciences. One view is that discriminatory glass ceilings cause these differences (Raggins, Townsend, & Mattis, 1999) or other social and psychological barriers (e.g., stereotype threat; Spencer, Steele, & Quinn, 1999). Another view is that while there are limited differences in average ability, the variability of individual differences among males is greater than among women, which leads to more men being represented at the highest end of the distribution (Hedges & Nowell, 1995). Another view is that differences in life choices among men and women cause the differences in performance (Ceci & Williams, 2010; Ferriman, Lubinski, & Benbow, 2009).

For people to reach their career goals, they must successfully pass through several barriers, and the selection of admitted applicants at many career stages is never solely based on objective ability and capacity to succeed. Unearned favorable opportunities can cause lasting advantages for members of favored groups (Merton, 1968), and the accumulation of these small advantages can then interact to create substantial differences in professional prospects and attainment (Martell, Lane, & Emrich, 1996). These biases are particularly problematic when success is defined by subjective evaluations by supervisors and peers. It is, therefore, essential that the performance of professionals is measured objectively, which is difficult to do within and especially across STEM domains.

Our approach to studying gender differences follows the expert-performance approach (Ericsson, 2006; Ericsson & Ward, 2007), where we identify domains with objective measures of performance. There are domains of performance requiring reasonably similar abilities to those necessary for success in professional STEM domains, which do not have most of the barriers associated with professional training and performance. This paper will study the competitive domain of SCRABBLE to assess if there are gender differences and, if so, to attempt to identify the determining factors. The domain of expertise in SCRABBLE is particularly interesting for research on gender because there are more female competitors than male. SCRABBLE also has an objective rating system based on the chess rating system designed by Elo (1965). Additionally, SCRABBLE has very few barriers to entering competitions because the fees in top tournaments are low—even the world championship has an entry fee of no more than £150 (Mind Sports International, 2014).

In this paper, we will discuss how our knowledge about the acquisition of expert performance during individuals’ lives may help us identify factors influencing gender differences in attained level of intellectual performance. We first review our current knowledge about gender differences in elite performance and associated differences in the distribution of individual differences in performance for females and males. We will then review evidence for individual differences in the development of performance resulting from particular types of experiences in the domain and domain-specific practice activities. This paper examines how differential engagement in activities may account for individual differences in attained performance in SCRABBLE and if such differential engagement might account for observed gender differences in attained performance.

## Gender differences in high levels of performance in domains emphasizing intellectual activity

There are domains emphasizing cognitive activity, such as chess, go, and bridge, where the best male players typically outperform the best female players to such a degree that the genders sometimes compete separately and some competitions are restricted to only females. Charness and Gerchak (1996) proposed that gender differences in chess could be accounted for by the vastly larger number of active male players in comparison to female players and thus might not reflect differences in ability between males and females. For example, even when the underlying ability distribution of the two genders is identical, one would find the average ability of the top 10 from a sample of 100,000,000 men would be much higher than that for the top 10 from a sample of 50,000 women.

Another explanation for gender differences in performance is that males are more motivated than females to perform well during competitions. For example, laboratory studies show that when a task is modified to increase competitive pressure, males improve their performance while females’ performance does not change significantly (Croson & Gneezy, 2009).

Finally, stereotype threat (Spencer et al., 1999) has been offered as an explanation of gender differences in chess performance. An experimental study by Maass, D’Ettole, and Cadinu (2008) found that women chess players who played matches against male players on a computer with the same chess ratings performed worse than their rating would predict when they were made aware that they were playing against a male opponent. This reduced performance is surprising because the participant’s chess ratings were based on tournament performance, that would have included many matches against males. Until research shows that female chess players perform worse when they play male players in tournaments, this explanation cannot account for gender differences in chess ratings and outcomes in chess tournaments.

Our review found only a few studies analyzing personality measures to account for gender differences in competitive performance. For instance, Duckworth and Quinn (2009) found no gender differences on the Grit Scale, which is hypothesized to measure perseverance and determination, when they analyzed an Internet sample and a sample of West Point cadets. Similarly, a study conducted at the National Spelling Bee found no differences between females and males in Grit or competition performance (Duckworth, Kirby, Tsukayama, Berstein, & Ericsson, 2011). Finally, Marsh et al. (2013) found no differences between genders for Harmonious Passion (passion that leads a person to choose to do what they love) or Obsessive Passion (rigid persistence in an activity) in an extensive analysis of 19 samples with a total of over 3500 participants.

## Gender differences in expert scrabble performance

In SCRABBLE tournaments, males perform at a higher level than females. The National Tournament divides players into six ranked divisions, and males dominate at the highest levels of performance. In 2002, 86% of competitors in the division with the best SCRABBLE players were male, while in the division with the lowest performance only 31% of competitors were male (McCarthy, 2008). Given that most players in SCRABBLE tournaments are female (Fatsis, 2001; Tierney, 2005), the higher ratio of female to male players cannot explain the male advantages in SCRABBLE—in fact, one would predict females should be more prevalent than men at the top level.

The gender distribution of high levels of SCRABBLE performance is similar to those in STEM fields where the predominance of males increases at higher levels of achievement (Lubinski & Benbow, 2006). Consequently, SCRABBLE is a domain with objective measures of performance and virtually unrestricted entry requirements, where many proposed general accounts of the gender gap described earlier are highly unlikely.

In a previous questionnaire research study on SCRABBLE tournament players, Halpern and Wai (2007) reported significantly lower SCRABBLE ratings for women with *d* = −0.26 (Study 1), *d* = −1.25 (Study 2), and *d* = −0.58 (Study 3). Consistent with male domination at higher levels, Study 2 had the highest average SCRABBLE ratings of the three studies and also a greater male advantage. There is some evidence that the gender differences in SCRABBLE are surprising. A recent survey of graduate students in a class on gender differences in cognition found that, on average, the students incorrectly predicted there would be more female than male champions (Halpern, Straight, & Stephenson, 2011), when in fact, the past ten World Champions in SCRABBLE were all males.

## The effect of experience on attained objective high-level performance

When we are interested in assessing potential gender differences in ability, it is essential that we study objective performance. In STEM fields, nearly all judgments of an individual’s achievement are made by predominately male individuals, such as male teachers, male administrators, or male peers (Brink & Benschop, 2014). Consequently, these males may be susceptible to making biased judgments of performance as well as bias in their support and encouragement of students and trainees. In this paper, we will adopt the expert-performance approach (Ericsson, 2006; Ericsson & Ward, 2007), where we focus on measures of objective performance.

In a review of factors associated with attaining expert levels of objective performance, Ericsson, Krampe, and Tesch-Römer (1993) found that acquisition of high levels of competitive performance requires engagement in extended teacher-guided practice activities specifically designed to improve current performance. (Ericsson, 2006, 2016). We refer to teacher-guided purposeful practice as deliberate practice, where the term purposeful practice refers to individualized practice with a particular goal, in which the individual engages in practice tasks with opportunities for feedback, repetition, and refinement (Ericsson & Pool, 2016). Deliberate practice requires that the individual meets regularly with a teacher, who can assess his or her current performance level, recommend appropriate targets for improvement and then describe training activities that the individual can engage in by themselves with opportunities for feedback, repetition, and refinement. Without the supervision of a teacher or coach, the individual must select practice activities in the absence of domain knowledge, often accumulated over centuries, that has proven effective in maximally improving performance.

Subsequent research has found that motivated young chess players study the recommended openings of chess games and attempt to select the best next move for particular chess positions (Charness et al., 1996, 2005; Ericsson et al., 1993). The latter activity meets the criteria for purposeful practice because the move selected by a player can be immediately compared to the best move generated by a chess computer. The player can repeat this move selection for many chess positions with immediate feedback to improve the planning processes mediating the selection of moves.

## Outline of studies

The current studies investigate the effects of gender in SCRABBLE within the framework of the acquisition and maintenance of expert performance. SCRABBLE was invented relatively recently in 1931 by Alfred Butts (National Public Radio, 2002), so there is much less knowledge about the nature of effective practice compared to other classic games with many centuries of history. In contrast to chess, the domain of SCRABBLE is a recently developed domain, and it lacks professional coaches and a large body of written knowledge about training. SCRABBLE players, therefore, must decide for themselves which types of training activities that they think are effective for improving their performance. Consequently, SCRABBLE players cannot engage in deliberate practice, but only purposeful practice and other types of practice.

There are two published studies of SCRABBLE tournament players and the relation between their performance and practice history. Halpern and Wai (2007, Study 1) estimated “the total number of hours the experts had practiced or played SCRABBLE” (p. 83) averaged 1904 h (SD = 2532). This combined estimate of hours of practice was significantly related to participants SCRABBLE rating (*r* = 0.22, *p* < 0.05), but this analysis did not evaluate the possibility that the benefits for improving performance from an hour of playing SCRABBLE might be different from an hour of solitary study specifically aimed at improving SCRABBLE skill. In an experimental study with elite and average SCRABBLE tournament players, Tuffiash, Roring, and Ericsson (2007) assessed separate estimates of the amount of studying and also playing SCRABBLE per week for every year since the start of playing SCRABBLE. For the most recent year of playing SCRABBLE, they also asked participants to estimate the number of hours that they had spent in different study activities per week. Tuffiash et al. (2007) found that elite players spent significantly more time studying (*M* = 11,128 h) than the average tournament players (*M* = 3696 h). The current study includes similar data from a larger representative sample than Tuffiash et al. (2007) to permit a more robust investigation of the effects of playing SCRABBLE games and practice alone (study) and their relations to gender differences. We will also control for variables that have been shown to predict skill in past studies such as current age, starting age, and age of the beginning of serious play (Campitelli & Gobet, 2008; Charness, Krampe, & Mayr, 1996).

## Study 1: gender differences in SCRABBLE skill while controlling for practice and other important variables

With our goal of understanding the factors determining differences between males and females’ SCRABBLE ratings, we will first determine if the hypothesized gender differences are significant. We will then evaluate whether skill differences due to gender will be significant even after controlling for individual differences in practice and other background factors. At the same time using a mediation analysis, we will test what variables explain a significant amount of the gender differences in skill.

### Procedure

#### Participants

During registration at the 2004 National SCRABBLE Championship tournament, participants were offered a survey along with an envelope with postage to allow it to be mailed back to the investigators. Participants were told that any received surveys would be entered into a raffle for a prize. We received surveys from 291 participants, and a total of 153 participants returned the survey to us. Of the returned surveys, 14 participants did not completely fill out the survey, and an additional six participants gave highly implausible answers, defined as reporting over 100 h of SCRABBLE-related activity per week or over 50 h of tournament play weekly. We restricted the current analysis to the remaining 133 surveys.

A subsequent analysis of the tournament listings showed that there had been 682 participants in the tournament. An examination of the players’ names and their pictures led to an estimation that 319 females and 363 males had participated. The response rate was higher among males than females *χ*^2^(1) = 7.05, *p* < 0.01.

Our final sample contained 48 females and 85 males. The sample was highly educated with the average years of education reported being equal to 17.22 (SD = 2.94). Education was unrelated to rating *r*(130) = −0.03, *p* = 0.08 or age *r*(130) = 0.01, *p* = 0.95. Each participant had a SCRABBLE rating, which is updated after each tournament and derived based on prior rating and tournament performance, previously developed for chess (Elo, 1965). Means and standard deviations for all variables used are given in Table 1.

**Table 1:** Summary of average SCRABBLE performance ratings, age-related variables, and time spent engaging in various practice activities for male and female participants in Study 1

Variable	Males	Females	Total
NSA rating	1470.26 (343.28)**	1226.23 (310.41)	1382.19 (350.91)
Age	44.87 (13.55)	54.62 (12.67)**	48.39 (14.00)
First tournament age	35.29 (12.58)	43.65 (11.56)**	38.31 (12.82)
Starting age	13.36 (9.33)	15.57 (9.05)	14.16 (9.26)
Cumulative serious play	3.37 (0.65)	3.50 (0.43)	3.42 (0.58)
Cumulative serious study	3.06 (0.86)*	2.90 (0.66)	3.00 (0.79)
Current SSSA	0.48 (0.34)*	0.35 (0.32)	0.43 (0.34)
Current GWS	0.56 (0.33)	0.52 (0.39)	0.54 (0.35)
Current SCRABBLE play	1.11 (0.32)*	0.96 (0.35)	1.01 (0.34)

Practice variables were log transformed (*N* = 133, 85 males)

*SSSA* SCRABBLE-specific practice alone, *GWS* General Word Study

* *t*>1.98, *p* < 0.05

** *t*>2.62, *p* < 0.01. Star is placed in the column with the statistically significantly higher value

#### Materials and procedures

The survey asked participants to estimate how many hours per week they had engaged in two activities for each year from birth to their current age as has been done in previous studies (Ericsson et al., 1993; Charness, Tuffiash, Krampe, Reingold, & Vasyukova, 2005). The two activities were serious study of SCRABBLE alone, and playing SCRABBLE seriously or studying SCRABBLE with others. An estimate for the number of hours of cumulative practice activities was calculated by multiplying the weekly estimates by 52 and adding the sums for all years (Côté, Ericsson, & Law, 2005; Ericsson et al., 1993). In a subsequent section of the survey, participants were asked about their current level of practice during a typical week. They were asked about how many hours per week the past year they engaged in several different activities (See “Appendix”). They also completed ratings of how effortful, enjoyable, and relevant to improving their skill these activities were. These ratings will be examined later in this article in the context of Study 2B. Only one item about practice was omitted from analysis. This item asked if they were currently taking lessons. Only 6 of 133 reported taking lessons, and 4 of them reported durations of 1 h or less. Finally, they were asked demographic questions about gender, current age, age they started playing SCRABBLE, and age they first played in a SCRABBLE tournament.

It is challenging to estimate the reliability of SCRABBLE ratings at the time of the tournament. To obtain an estimate we capitalized on the fact that there were two segments in the competition (16 games and 15 games respectively). The correlation between the ratings for the two segments was *r*(120) = 0.84, *p* < 0.001.

#### Plan for data analysis

First, we estimated the overall effects of gender on SCRABBLE rating. Then we analyzed practice activities and how to best form composite measures of them in a hierarchical multiple regression—consistent with past studies of purposeful and deliberate practice. The main analysis involved a mediation analysis of the relationship of gender and SCRABBLE ratings through the covariates of the composite variables measuring practice, first tournament age, starting age, and age. Our analysis uses the indirect method of Preacher and Hayes (2008) which uses the bootstrap method to calculate 95% confidence intervals for the indirect effects.

### Results

Being female was significantly negatively correlated with SCRABBLE ratings, *r*(131) = −0.35, *p* < 0.001 (Supplementary Online Materials, Table 1, subsequently referred to as Table SOM1), which corresponds to *d* = −0.74. Gender explained around 12% of the variance in SCRABBLE ratings.

A complete correlation table with all variables is reported in Table SOM1. Cumulative SCRABBLE study alone and cumulative serious play were significantly related to SCRABBLE ratings. Current practice activities were described by several variables and could be best accounted for by three variables: SCRABBLE play, general word study (studying dictionaries and word lists), and SCRABBLE-specific practice alone (anagramming and analyzing SCRABBLE games). Our “Appendix” describes which questions provided information for the three groups of practice activities. For a validation of dividing current activity into three variables see SOM-A1 and Table SOM2. We also conducted a hierarchical multiple regression analysis predicting SCRABBLE ratings (see details reported in SOM-A2 and Table SOM3).

Finally, we conducted a mediational analysis of the relation between gender and SCRABBLE ratings (for detailed results see Table 2). When all predictor variables were tested as mediators of gender effects using the bootstrap method (Preacher & Hayes, 2008) we found that three variables had significant indirect effects. Age had an indirect effect tending to diminish the effect of gender. First tournament age had an indirect effect that tended to increase the effect of gender. Finally, current SCRABBLE-specific practice alone also had a significant indirect effect whereby SCRABBLE-specific practice alone tended to decrease the effect of gender. The set of variables included in the analysis predicted 64% of the variance in SCRABBLE rating. All variables statistically significantly related to rating in the final model except cumulative SCRABBLE study, including gender. It is particularly noteworthy that the amount of current general vocabulary study negatively related to rating. We should note that there was no evidence of a response bias in the estimates of females in comparison to males on their time inventories. An analysis of the two cumulative variables does not show a significant between-subject gender difference *F*(1,133) ≤0.01, *p* = 0.99, and *F*(1,133) = 0.03, *p* = 0.88, with males being insignificantly higher on both.

**Table 2:** Mediation analysis of the relationship of gender and rating through experience variables and age (*N* = 133, 85 males)

Path	*B* (95% CI)	*t* score
Direct paths		
Gender to rating	170.86 (−257.75, −83.97)	−3.89**
Cumulative serious play to rating	185.44 (95.80, 275.08)	4.10**
Cumulative study alone to rating	55.44 (−0.94, 111.82)	1.95
Current SCRABBLE play to rating	62.84 (−76.59, 202.27)	0.89
Current gen word study to rating	−199.18 (−320.48, −77.89)	−3.25**
Current SCRABBLE study alone to rating	167.73 (30.64, 304.84)	2.42*
Age to rating	5.98 (0.06, 11.90)	2.00*
Starting age to rating	4.93 (0.22, 9.63)	2.07*
First tournament age to rating	−18.24 (−24.30, −12.18)	−5.96**
Indirect gender to rating		
Through cumulative serious play	24.27 (−6.77, 64.02)	
Through cumulative study alone to rating	−8.53 (−42.48, 3.76)	
Through current SCRABBLE play to rating	9.39 (−7.87, 39.70)	
Through current gen word study to rating	7.75 (−18.35, 35.89)	
Through current SCRABBLE study alone to rating	−22.92 (−60.82, −3.71)	
Through age to rating	58.32 (7.13, 143.42)	
Through starting age to rating	10.88 (−4.00, 34.56)	
Through first tournament age to rating	−152.32 (−255.96, −74.29)	

* *p* < 0.05, ** *p* < 0.01

### Discussion

Our study replicated and greatly extended the findings reported by Halpern and Wai (2007). We found a significant difference in SCRABBLE ratings between men and women (*d* = −0.74), replicating Halpern and Wai (2007) findings of effects ranging from *d* = −0.26 (Study 1) to *d* = −1.25 (Study 2). Halpern and Wai (2007) collected data on the estimated life-long accumulation of hours of SCRABBLE play and study only in Study 1. This study found a significant correlation (*r* = 0.22, *p* < 0.05), which explains only 5.0% of the variance of the SCRABBLE ratings.

One of the reasons was that our study represents practice in a much more complex way. One of the most interesting findings of Study 1 is that the effect on SCRABBLE skill of engagement in different study activities conducted alone differs as a function of the particular type of activity performed alone. In fact, the hours of studying vocabulary alone was a negative predictor of SCRABBLE skill, whereas solitary practice analyzing SCRABBLE games and practicing anagrams were strong positive predictors of SCRABBLE skill.

Asking participants to report a single overall estimate for practice alone for each year would provide a sum of these durations, and thus could not predict attained SCRABBLE ratings as accurately as possible. In fact, although the cumulative amount of practice alone was found to be correlated with SCRABBLE rating in our sample (See Table SOM1), this variable does not predict significant independent variance in SCRABBLE ratings as seen in Table 3. The negative regression weight for the log-transformed amount of general word study should not be interpreted as an active cause of lower SCRABBLE performance. In fact, the zero-order correlation between SCRABBLE skill and log-transformed amount of general word study is not significantly different from zero *r*(131) = −0.09, *p* = 0.16. An alternate explanation of the negative prediction of rating is that the amount of estimated study time for general vocabulary serves as a suppressor variable because there is a correlated bias in our estimates of the amount of practice activity (Conger, 1974). In this case, the general word study variable would either be unrelated to skill acquisition, or possibly very slightly harmful. If general word study is unrelated to skill acquisition, it would still share irrelevant information with our other practice variables. By allowing unrelated variance to be statistically controlled, it would reveal a stronger relationship between purposeful practice and skill—closer to their true relationship.

**Table 3:** Summary of SCRABBLE ratings, demographic variables, and estimated duration in various practice activities collected in Study 2A

Variable	Males	Females	All
NSA rating	1410.15 (291.98)**	1215.21 (269.44)	2.67 (0.66)
Age	44.69 (14.39)	55.67 (12.31)**	50.36 (14.40)
Starting age	12.37 (8.54)	13.62 (8.70)	13.02 (8.61)
First tournament age	34.97 (13.28)**	42.35 (11.40)	38.78 (12.84)
Log play vs others	3.77 (0.56)	4.00 (0.37)**	3.89 (0.48)
Log play vs computers	2.88 (1.66)	2.29 (1.67)	2.59 (1.68)
Log analyze own games	2.52 (1.23)**	1.76 (1.58)	2.12 (1.47)
Log analyze other games	1.54 (1.42)	1.11 (1.46)	1.32 (1.45)
Log study anagrams	2.94 (1.38)*	2.28 (1.59)	2.60 (1.52)
Log play word games	1.33 (1.62)	1.95 (1.72)*	1.65 (1.69)
Log play self	0.89 (1.52)	0.99 (1.55)	3.80 (0.48)
Log study definitions	2.25 (1.36)	2.25 (1.51)	2.25 (1.43)
Log study spelling	2.55 (1.44)	3.04 (0.95)*	2.81 (1.23)
Log create word list	2.48 (1.42)	1.95 (1.56)	2.20 (1.51)
Log scrabble tournaments	3.03 (1.11)	2.66 (1.47)	2.84 (1.16)

All practice variables were log transformed (*N* = 122, males = 59)

* *t*>1.98, *p* < 0.05, ** *t*>2.61, *p* < 0.01. Star is placed in the column with the statistically significantly higher value

Our study found large gender differences in SCRABBLE skill that are comparable to Halpern and Wai (2007). The primary goal of our study was to examine if females’ SCRABBLE skill would remain systematically lower than the males even after controlling for all differences in experience, practice, and career trajectories. We found that after the statistical control of these other factors the gender differences remained significant.

The mediation analysis shows that there are multiple indirect effects of gender on rating. Two of the demographic variables seemed related to the gender differences in SCRABBLE skill. The first is age, females were on average in this sample older, and that was associated with diminishing the gender difference. Females also began tournament play at a later age and this difference was associated with increased gender differences in ratings. However, the fact that we were unable to estimate all variance attributable to practice raises the possibility that with more detailed data on past practice a larger portion of the gender differences would be accounted for, perhaps all reliable gender differences might be ultimately explained. This is particularly possible since current SCRABBLE-specific practice alone appears to significantly decrease the effect of gender according to the mediation analysis.

## Study 2: accounting for gender differences by differential involvement in practice

Study 1 showed that after controlling for practice, experience, and starting age, the effect of gender on SCRABBLE ratings was reduced from a large advantage to a medium-sized advantage favoring males. We also found that all of the time spent in SCRABBLE-related activities conducted alone may have met the criteria for purposeful practice, yet the amount of engagement in them differentially influenced attained performance. Consequently, if we could collect estimates of engagement in particular types of practice alone, we might be able to find that differential engagement in practice activities by males and females might account for the gender differences in attained SCRABBLE skill.

Without a valid body of knowledge about the differential effects of different types of practice activities for improving SCRABBLE performance, there could be no purposeful practice designed and guided by teachers and coaches (cf. the definition of deliberate practice as described in Ericsson et al. (1993) and Ericsson and Pool (2016)). In SCRABBLE, the best available approach to finding effective purposeful practice involves drawing analogies from other domains of expertise. In line with previous research on expert performance in chess and music, we would predict a significant effect of the amount of time spent on practice activities conducted alone. Consistent with the hypothesis that duration of engagement in practice activities designed to improve domain-specific performance mediates skill acquisition, we found in Study 1 that analyzing actual SCRABBLE games and training re-arranging letter sequences into words (anagram problem solving) were significant positive predictors of SCRABBLE ratings. In contrast, the correlation between vocabulary study and SCRABBLE rating was non-significant before controlling for other variables.

In Study 2 we asked separate questions on engagement in each of the different types of practice-alone activities, hypothesizing that these data would successfully account for most of the gender differences in SCRABBLE ratings. If this were the case, we need to be able to answer a new question: why would females prefer to engage in practice activities that are less effective than those engaged in by men? We thus separated the analyses of Study 2 into two parts. The first part focused on accounting for gender differences in SCRABBLE ratings by differential engagement in practice activities. The second part analyzed ratings of different practice activities and whether any differences in personality account for the differential preferences of female players for engaging in effective practice activities.

## Study 2A: gender and age differences in skill with better control for practice

To test the hypothesis that differential engagement in practice activities can account for gender differences in attained SCRABBLE skill, we conducted a second empirical study with a revised questionnaire. Study 2A collected estimates of engagement in a more varied set of possible SCRABBLE-related activities that could differentially influence the development of skill. These activities included SCRABBLE-specific practice alone, general word study, and playing/studying SCRABBLE with others, along with several other activities (see Table 4 for a complete list with means and standard deviations). We asked participants to recall their current engagement in this new set of activities (as was done in Study 1 for the three activities). We also asked them to recall their engagement in these activities during the first year they played SCRABBLE and during the year of their highest SCRABBLE skill (NSA rating). By averaging these three values for each activity and multiplying the average by the number of years they had participated in the domain, an estimate of the cumulative amount of this activity across their career was derived. This estimate was then given a logarithmic-transformation before the data analysis. The primary goal of Study 2A was to develop a method of measuring the engagement in different types of experiences and practice activities so we would have a rich and varied set of factors that can predict the current level of SCRABBLE skill. We hypothesized that this method would permit us to develop a better set of measures of practice activities, which would permit statistical control of individual differences between men and women in their engagement in practice and experience related to SCRABBLE.

**Table 4:** Goodness-of-fit indicators of models for practice activities in SCRABBLE and the structural equation model (SEM) for relation between practice and demographic variables with scrabble skill (*N* = 122)

Model	*df*	*χ* ^2^	*χ*^2^/*df*	SRMR	TLI	CFI	RMSEA
Single factor	44	83.70**	1.90	0.086	0.67	0.74	0.086
Two factors	42	81.21**	1.93	0.086	0.66	0.74	0.088
Three factors**	40	65.35**	1.63	0.077	0.77	0.83	0.072
Four factors**	38	46.44	1.22	0.064	0.92	0.94	0.043
Five factors*	35	38.33	1.10	0.057	0.97	0.98	0.028
SEM	65	96.16**	1.48	0.062	0.87	0.93	0.063

*SRMR* standardized root mean residual, *TLI* Tucker–Lewis index, *CFI* comparative fit index, *RMSEA* root mean square error of approximation

* *p* < 0.05 and ** *p* < 0.01. For model * *p* = < 0.05 and ** *p* < 0.01 for improvement of fit compared to previous model based on change in Chi square

### Methods

#### Participants

At the National SCRABBLE tournament in 2008 (four years after the 2004 national tournament studied in Study 1), 260 participants were handed the questionnaire. 148 participants returned our questionnaire and were entered into a raffle for a prize. After 26 participants’ questionnaires had been eliminated by the same criteria as in Study 1, 122 questionnaires were submitted to the analyses. The average age of this sample was 50.36 years with SD = 14.40. The average starting age for playing SCRABBLE was 13.02 with SD = 8.61. The average age at the first participation in a SCRABBLE tournament was 38.78 with SD = 12.84. Our sample contained 63 females and 59 males. At the tournament, there were 312 females and 350 males according to our hand coding of names and pictures suggesting similar response rates *χ*^2^(1) = 1.01, *p* = 0.31. The improved response rate of women was likely due to the third author making a more explicit effort to recruit females given the disproportionate response rates and gender differences noted in Study 1 and the disproportionate response rates. Again the sample was highly educated reporting 18.13 (SD = 2.91) years of education, which again was unrelated to rating *r*(118) = −0.03, *p* = 0.75, or age *r*(120) = 0.01, *p* = 0.96. The average SCRABBLE rating was 1309.48 with SD = 296.03.

#### Materials

The questionnaire used in Study 2A differed from the previous questionnaire used in Study 1. The current questionnaire asked about the estimated engagement in more specific practice activities. To answer all questions about activities for every year of their life would take a great deal of time; consequently, participants were asked to make their estimates for three time points in their careers, namely at the current time, at the time of their highest SCRABBLE skill rating, and the year when they started playing SCRABBLE.

To assess the relation between our new method used in Study 2A and the traditional method eliciting retrospective estimates of engagement in each activity for each year, we conducted a few preparatory analyses on data from other studies which had collected yearly estimates for every year of participation. Based on data on practice alone in a chess study by Charness et al. (2005), we calculated our new measure based solely on data from 3 years: the first year of participation, the current year, and the year of highest chess rating during each participant’s career. Our new measure was based on only three highly correlated estimates [*r*(348) = 0.81, *p* < 0.001], with the estimate based on the aggregation of data from each year of the chess players’ careers. We also conducted an analysis of the data set from our Study 1. There were 125 participants in Study 1 who reported data on their best year, starting year, and current year so we could calculate the estimates used for cumulative practice alone in Study 1 and via the new method. The correlation between the two measures was *r*(123) = 0.73, *p* < 0.001. The relatively high correlation between data collected with the old method used in Study 1 and our new method supported our decision to use the new abbreviated method. Again information from the item of taking lessons in SCRABBLE was omitted because only three out of 122 participants reported currently doing that and all three reported less than an hour a week.

#### Outline for the data analysis

Our analysis follows the same general structure as the one reported in Study 1. First we estimated the gender differences by comparing the SCRABBLE skill of males and females without controlling for any other variables. Then, we performed a confirmatory factor analysis (CFA) to assess the structure and relations between the large number of variables measuring practice. The goal was to confirm that a unitary model of practice is not appropriate, as we argued in Study 1, and, more importantly, to identify the most accurate measurement model possible to predict skill from the variables measured in our dataset. Once we identify the factor structure of our measured practice variables, we will use a structural equation model to test how the best practice model predicts skill and how the demographic factors can account for SCRABBLE skill over and above practice. Based on our findings in Study 1, we predict that among the practice variables, only increases in SCRABBLE-specific study alone will be associated with increases in SCRABBLE skill, while increases in general word study will be associated with decreases in skill. We predict that with this more detailed description of practice activities, experience, and career history with SCRABBLE, the effects of gender might no longer be significant. We will also replicate the mediation analysis using the sum scores identified by factor analysis to assess if the pattern is similar to that found in Study 1. Our analysis will be based on scores summed across variables measuring the same type of practice variables (as determined by the factor solution from the CFA) to avoid potential issues with known problems of indeterminacy of estimated factor scores.

To confirm that practice is not a unitary construct, we will run three confirmatory factor analyses. However, given the main interest is gender differences, it is important to have a strong measurement model as the basis for the structural equation model. Therefore, if variables have a low extraction (less than 0.2) after the three-factor solution, those variables will be grouped together for an additional factor or allowed to be independent if necessary.

### Results

We first examined correlations between demographic variables and SCRABBLE rating to assess whether the pattern from Study 1 was replicated. The correlation between being female and rating was −0.33 (*p* < 0.01) compared to −0.35 (*p* < 0.01) in Study 1. The mean SCRABBLE rating for males was 1410.15 (SD = 291.98) compared to 1215.21 (SD = 269.44) for females, *d* = −0.69 thus replicating the earlier observed gender differences, and again being among the largest gender differences studied in cognitive psychology. The zero-order correlations of the participants’ gender, SCRABBLE ratings, first tournament age, and current age are reported in Table SOM2. Table 4 shows the means for all variables for both males and females.

**Fig. 1:**
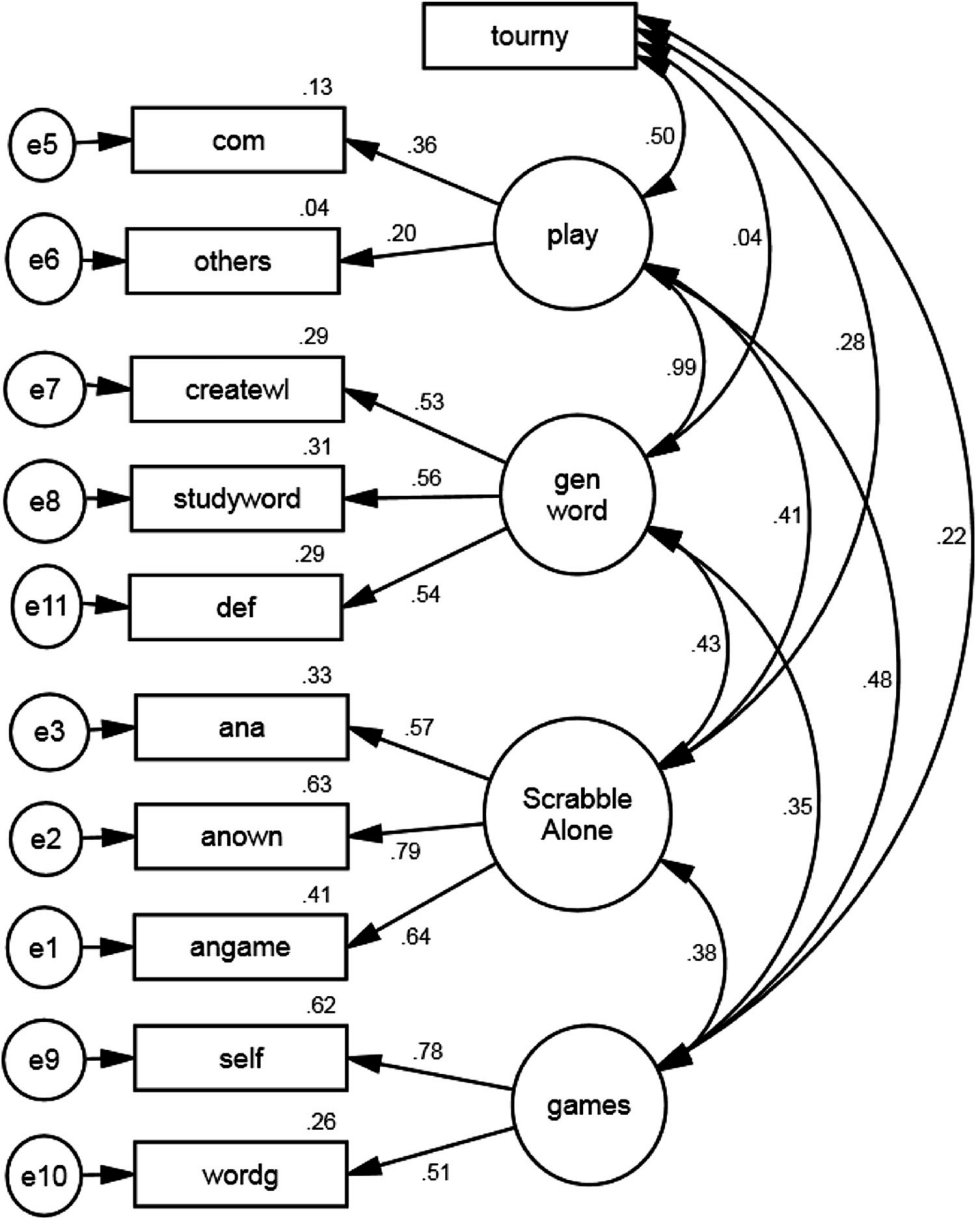
CFA results. *Angame* analysis games of others, *anown* analyze own games, *ana* study anagrams, *def* study definitions, *studyword* study word list, *createwl* create world list, *wordg* play word games other than *SCRABBLE, SELF* play SCRABBLE against self, *others* plays SCRABBLE against others, *com* play against computer, *tourny* play in a SCRABBLE tournament

All variables measuring practice activities and other types of domain-related activities in SCRABBLE were submitted to a CFA. The results from the CFA are displayed in Table 5 and Fig. 1. The single-factor model assumes that all practice reflects the same construct and had a good fit to the data by the *χ*^2^/*df* and SRMR, while the TLI, *χ*^2^, CFI, and RMSEA suggested an inadequate fit. The two-factor model involved splitting practice into either practice alone, on the one hand, or playing SCRABBLE and practicing with someone else, on the other, failed to improve on the one-factor model *χ*^2^(2) = 1.40, *p* > 0.05. The three-factor model involved separating practice alone into two types, namely SCRABBLE-specific practice alone and general vocabulary study, and this model had a better fit than both the two-factor model with *χ*^2^(2) = 15.86, *p* < 0.001 and the one-factor model *χ*^2^(4) = 18.35, *p* < 0.01. For the three-factor model, *χ*^2^/*df* and SRMR suggested a good fit to data, the RMSEA a moderate fit, but the TLI, *χ*^2^, and CFI suggested inadequate fit. The statistically significant improvement in the model fit supports the hypothesis that all types of practice alone do not represent the same theoretical construct. Given our main interest is to mediate gender differences, to improve the fit of the model, we generated a four-factor model by creating a new variable measuring engagement in games that were not part of actual SCRABBLE competitions, including other word games and games against oneself. This model also improved on the model fit over the three-factor model *χ*^2^(2) = 18.91, *p* < 0.001 given a good fit for all measures except for the TLI and CFI, which suggested some misfit. Finally, we generated a five-factor model where playing in a SCRABBLE tournament was considered a separate factor because this activity differs from other types of SCRABBLE play. This model also significantly improved model fit *χ*^2^(3) = 8.1, *p* < 0.05 over the four-factor model and gave good or better fit to data on all measures of model fit.

**Table 5:** Summary of the direct effects of latent practice variables and demographic variables on SCRABBLE skill in the structural equation model

Variable	*B*	SEM	*β*	*R* ^2^
				0.51**
Log SCRABBLE tournaments	0.06	0.08	0.08	
Latent SCRABBLE alone	0.51	0.19	0.46**	
Latent SCRABBLE play	−0.04	0.24	−0.01	
Latent General Vocab Study	−0.72	0.34	−0.45*	
Latent games	0.01	0.09	0.02	
Starting age	−0.25	0.12	−0.25*	
First tournament	−0.48	0.14	−0.48**	
Age	0.39	0.17	0.39*	
Gender	−0.29	0.20	−0.15	

Age variables and rating were *z* scored to minimize computation. Females are given a higher value than males for the gender variable (*N* = 122)

* *p* < 0.05 and ** *p* < 0.01

When the demographic variables were added to the five-factor model and a structural equation model was tested predicting skill, the resulting final model had a good fit to the data with the *χ*^2^/*df* and SRMR, the RMSEA a moderate fit for the TLI, while *χ*^2^ and CFI suggested an imperfect fit. Overall, the fit suggests that we have an interpretable model as shown in Table 6 (see Fig. 1). In our final model, current SCRABBLE skill was predicted by starting SCRABBLE at a younger age, starting SCRABBLE tournament play younger, and being older. When practice and experience are accounted for, the duration of involvement in SCRABBLE benefits skill, whereas current age is not associated with a decline of skill. Consistent with the pattern observed in Study 1, SCRABBLE practice alone positively predicted skill and general word study negatively predicted skill; no other study variables were significantly related to skill.

**Table 6:** Mediation analysis of the relationship of gender and rating through experience variables and age (*N* = 122)

Path	*B* (95% CI)	*t* score
Direct paths		
Gender to rating	−116.74 (−217.11, −16.36)	−2.30*
Games played to rating	−11.77 (−38.71, 15.17)	−0.87
SCRABBLE play to rating	−9.95 (−38.99, 19.09)	−0.68
SCRABBLE-specific study alone to rating	48.74 (11.33, 86.15)	2.58*
General word study to rating	−40.03 (−96.33, 16.27)	−1.41
SCRABBLE tournament study to rating	45.18 (8.57, 81.78)	2.45*
Age to rating	6.6 (1.24, 11.96)	2.44*
Starting age to rating	−3.65 (−9.19, 1.89)	−1.31
First tournament age to rating	−11.67 (−17.38, −5.95)	−4.05**
Indirect gender to rating		
Through games played to rating	−7.66 (−39.79, 9.11)	
Through SCRABBLE play to rating	−8.18 (−47.62, 15.32)	
Through SCRABBLE-specific study alone to rating	−25.4 (−73.03, −1.85)	
Through general word study to rating	−1.74 (−22.69, 12.52)	
Through SCRABBLE tournament study to rating	−16.99 (−59.88, 0.38)	
Through age to rating to rating	72.43 (15.63, 169.78)	
Through starting age to rating to rating	−4.55 (−28.54, 4.49)	
Through first tournament age to rating	−86.13 (−184.54, −32.42)	

* *p*<0.05, ** *p*<0.01

Most interestingly, our final model showed no significant relation between gender and skill. To better understand how we explained the gender effect, we examined the partial co-variances within the model. According to this model females were found to study SCRABBLE alone significantly less than males (*p* < 0.01), but again this did not reflect an overall gap in domain engagement, as females played non-tournament SCRABBLE games significantly more (*p* < 0.01) than males. Factor loadings for this model are in Table SOM5.

Finally, the mediation analysis from Study 1 was replicated using the variables in Study 2A. In this analysis, to be consistent with Study 1, sum scores were generated based on the five-factor model identified previously. This analysis found the same pattern as in Study 1, with age having a positive indirect effect of *b* = 72.43 with 95% CI (15.90, 155.85), and where age also significantly increased the effect of gender. Tournament age had a negative indirect effect of *b* = −86.12 with 95% CI (−168.62, −29.99), where first tournament age significantly accounted for the lower SCRABBLE ratings of females. Finally, SCRABBLE-specific practice alone had a significant indirect effect *b* = −25.40 with 95% CI (−73.66, −3.39), where increased SCRABBLE-specific practice alone by males significantly accounted for the lower ratings of females. Consistent with Study 1, no other variables were significantly related to gender differences in performance. The detailed results are reported in Table 6. There was no evidence of a general response bias in the reporting of hours in males compared to females on their time inventories. An analysis of the combined gender effect of the five practice activities shows no overall gender difference *F*(1,120) = 0.64, *p* = 0.43 with females reporting an insignificantly larger amount.

### Discussion

When the effects of our more refined measures of experience and practice activities were statistically controlled in Study 2A, we found no significant residual effect of gender. The negative zero-order correlation between female gender and SCRABBLE skill is likely attributable to less engagement in SCRABBLE-specific practice alone over the course of the lifetime and more engagement in other activities that give less benefit for improving SCRABBLE skill. In Study 2A we were able to trace the differences in types of practice during the entire developmental career, and after statistical control for demographic and practice variables, the residual relation to gender was reduced to below statistical significance (*p* = 0.15). It is essential to measure separately the engagement in different practice activities during the development of SCRABBLE skill to identify and measure differential engagement by females and males. We did not find any differences in overall engagement in SCRABBLE-related activities. Both Study 1 and Study 2A found types of activities where females are more engaged and types of activities where males are more engaged. Of the activities we measured, females had spent significantly more time in three of these activities whereas males spent more time in three of the other activities (see Table SOM4).

In Study 2A we were more able to account for the role of estimated accumulated SCRABBLE-specific practice than in Study 1, where the participants gave a single estimate for practice alone that included both SCRABBLE-specific practice and general vocabulary learning. We are confident that our findings in Study 2A are consistent with the findings from Study 1. When we regressed the accumulated estimates of all types of practice alone in Study 1, the standardized regression weight for cumulative practice alone was 0.12, *p* > 0.05; in contrast, the corresponding regression weights for cumulative SCRABBLE-specific practice was 0.46, *p* < 0.001 and for cumulative general word study was −0.45, *p* < 0.001. As skill increases, players engaging in practice activities directly related to SCRABBLE spend relatively less time building their vocabularies, as demonstrated in both concurrent activities in Study 1 and cumulative estimates in Study 2A. While the factor analysis identified five different independent factors to describe the shared variance in engagement in many SCRABBLE-related activities, only two of these factors were statistically significantly related directly to SCRABBLE skill in our Study 2A model. These were the two types of practice identified in Study 1, with general vocabulary study (an activity that is done alone) being negatively related to skill and SCRABBLE-specific practice alone being positively related to skill.

## Study 2B: gender differences in subjective ratings of practice activities and personality tests

Individual differences in the engagement in SCRABBLE-specific practice alone and starting age were able to account for all of the statistically significant differences in SCRABBLE ratings attributable to gender in Study 2A. The lower SCRABBLE ratings for women could be accounted for by women’s decisions to engage more in practice activities where increased engagement was not related to effective improvement of SCRABBLE performance. To better understand these differences, we analyzed additional data collected for Study 2 in the last part of the questionnaire. We collected data on the participants’ subjective perceptions of the relevance, enjoyment, and effort of a set of practice activities. After the questionnaire, we asked participants to fill out several additional personality measures that Duckworth et al. (2011) had found to be related to purposeful practice in preparation for competitions in spelling, such as Harmonious and Obsessive Passion (Vallerand et al., 2007) as well as Grit (Duckworth & Quinn, 2009). By collecting information on all of these additional personality characteristics, and subjective perceptions of various domain-related activities, we attempted to uncover factors that might account for gender differences in engagement in alternative types of practice activities with differential effects on improving SCRABBLE skill.

### Method

#### Participants

Nearly all of the 122 participants in the analysis for Study 2A completed the personality tests. All but one participant answered the Grit questionnaire and all participants answered the Harmonious and Obsessive Passion questionnaire. All participants answered at least one question about the relevance of each category of practice, 114 answered the questions regarding enjoyment of practice, and 115 answered the questions about the effort associated with various practice activities. Because the largest differences between the participants answering different questions was *t* = 0.25, *p* = 0.80 for age, and *χ*^2^ < 0.01, *p* = 0.96 for gender, we felt comfortable treating the data as having values missing at random and used multiple imputations using 10 imputations to replace the missing data (Scheffer, 2002).

#### Materials and procedures

In the last part of the questionnaire, the participants were asked to rate a set of SCRABBLE related activities on scales of 0–10 according to their relevance for performance improvement, the amount of effort, and intrinsic enjoyment. This type of rating has been collected in research on deliberate practice from its beginning to provide perceived characteristics of practice alone in music (Ericsson et al., 1993). In Study 2A we identified the 5 latent factors distinguishing different activities. If a participant answered at least one question for a given variable for any of the rated activities, we then assigned that score or an average score based on all available ratings for the associated variable.

#### Data analysis

The mean ratings of relevance, enjoyment, and efforts for practice activities as a function of gender are given in the Table SOM6, and the mean scores for the personality tests are given in Table 7. First, we analyzed the ratings of the practice activities with a repeated-measure ANOVA to assess whether the ratings differed between the practice activities. We then used hierarchical logistic regression to identify individual differences that were related to gender. In the final step, we conducted analyses that only included variables as mediators that had contributed unique significant variance to prediction of the target variables, namely SCRABBLE skill and gender. We applied the bootstrapping method (Preacher & Hayes, 2008). These variables were examined in mediation models relating gender and engagement in current practice activities.

**Table 7:** Summary of ratings of various personality measures used in Study 2B (*N* = 122)

Variable	Males	Females	All
Grit	2.77 (0.72)	2.58 (0.57)	2.67 (0.66)
Harmonious passion	5.18 (1.01)	5.44 (1.03)	5.31 (0.99)
Obsessive passion	2.83 (1.07)*	2.39 (1.24)	2.60 (1.14)

* *t*>1.98, *p* < 0.05, ** *t*>2.61, *p* < 0.01. Star is placed in the column with the statistically significantly higher value

### Results

#### Analyses of subjective ratings as a function of practice activity

In a repeated-measures ANOVA, participants’ ratings of the relevance to improvement of different practice activities were found to differ significantly from each other *F*(3.51,425.21) = 96.13, *p* < 0.001. Bonferroni-corrected post hoc testing of mean differences between all pairs of practice activities (Table 8) showed that tournament play was rated as significantly more relevant for improvement than all other activities. Players rated playing SCRABBLE as more relevant than any activity other than tournament play. Playing other types of word games was rated as significantly less relevant than all other activities.

**Table 8:** Summary of post hoc comparisons of different practice activities

Comparison	Relevance	Effort	Enjoyment
SCRABBLE-specific practice alone vs tournament play			
	7.90 (−1.44)*	15.78 (−2.87)*	16.94 (−3.08)*
SCRABBLE-specific practice alone vs SCRABBLE play			
	4.11 (−0.74)*	7.84 (−1.43)*	13.36 (−2.43)*
SCRABBLE-specific practice alone vs games			
	8.37 (1.52)*	2.20 (−0.40)	6.34 (−1.15)*
SCRABBLE-specific practice alone vs general word study			
	0.84 (−0.15)	0.18 (0.03)	1.44 (−0.26)
Tournament play vs SCRABBLE play	5.84 (1.06)*	11.82 (2.15)*	6.01 (1.09)*
Tournament play vs games	20.23 (3.68)*	14.46 (2.63)*	12.62 (2.29)*
Tournament play vs general word study	7.89 (1.43)*	15.76 (2.86)*	16.23 (2.95)*
SCRABBLE play vs games	17.14 (3.12)*	7.38 (1.34)*	10.22 (1.85)*
SCRABBLE play vs general word study	3.66 (0.67)*	8.14 (1.48)*	13.71 (2.49)*
Games vs general word study	9.53 (−1.73)*	2.17 (0.30)	4.86 (0.88)*

The first value reflects the *t* value of the test the number in parenthesis represents Cohen’s *d* (*N* = 122)

* At the Bonferroni critical *p* = 0.005

The repeated-measures ANOVA of inherent enjoyment found that practice activities differed significantly in their rated inherent enjoyment *F*(3.38,408.52) = 134.56, *p* < 0.001. Bonferroni-corrected post hoc tests of all pairs of activities (see Table 8) showed that tournament play was rated as more enjoyable than all other activities. Playing SCRABBLE was rated as more enjoyable than all activities except tournament play. Playing other types of word games was rated as more enjoyable than SCRABBLE-specific practice alone and general word study.

Finally, ratings of effort significantly differed for the practice activities, *F*(3.42, 414.09) = 86.67, *p* < 0.001. Bonferroni-corrected post hoc tests (see Table 8) showed that tournament play was rated as more effortful than other activities. Playing SCRABBLE was rated as more effortful than all other activities except for tournament play.

#### Ratings and personality as a function of gender

The 15 subjective ratings of activities and the three personality measures were significantly associated with gender *χ*^2^(18) = 41.04, *p* < 0.01, but only ratings of inherent enjoyment significantly predicted unique variance *χ*^2^(5) = 15.93, *p* < 0.01, as shown in Table 9. A regression analysis involving only the ratings of enjoyment showed that females rated SCRABBLE-specific practice alone less enjoyable than males, while they rated playing SCRABBLE outside of tournaments more enjoyable than males. No other effects were significant. This finding suggests that enjoyment ratings are also relevant to discriminating between males and females. The same set of variables was used to predict SCRABBLE skill instead of gender. Again enjoyment ratings were the only significant predictors (See Table SOM7).

**Table 9:** Summary of hierarchical logistic regression analysis of rating of various practice activities for relevance, effort, and enjoyment entered as a set predicting gender

Variable	*χ*^2^ total	*χ*^2^ unique	*B*	SEM	*p*	*χ* ^2^
Relevance	8.91	2.70				
Joy	27.03**	15.93*				
Effort	18.42**	5.95				
Personality	6.32	5.50				
Final logistic regression						27.03
Scrabble tournament play joy			−0.18	0.15	0.24	
Playing scrabble joy			0.52	0.18	<0.01	
General vocabulary joy			0.18	0.11	0.12	
Playing games joy			−0.22	0.13	0.11	
SCRABBLE-specific practice alone joy			−0.39	0.13	<0.01	

At the bottom the multiple regression analysis is shown for enjoyment ratings, which was the only set of variables that significantly improve model fit in a unique manner (*N* = 122)

* *p* < 0.05 and ** *p* < 0.01

#### Mediating engagement in practice as a function of gender differences using enjoyment ratings

We hypothesize that enjoyment ratings predict both skill and gender. However, the enjoyment of particular practice activities should not directly change SCRABBLE skill but instead should influence engagement in relevant practice activities and that engagement would change performance. If females enjoy SCRABBLE-specific practice alone less than males (as shown in Table 9), that might explain why they spend less time in that activity as shown in Study 1 and Study 2A. A mediational analysis of the relation between gender and amount of accumulated SCRABBLE play found a direct effect of gender on SCRABBLE play (*p* = 0.02), where females played SCRABBLE for more hours. The mediational analysis of the relation between gender and hours of engagement in SCRABBLE-specific practice alone found no direct effect of gender on the amount of SCRABBLE-specific practice alone (*p* = 0.32). However, the indirect path via enjoyment was significant (*p* = 0.01), with lower values of enjoyment for females. This satisfies the criterion for full mediation (Table 10).

**Table 10:** Mediation analysis testing if gender differences in subjective rating of activities or personality mediate gender differences in actual participation in the activity

Practice variable	Path	B	SEM	*t* score	*Z* score
SCRABBLE-specific practice alone					
	Gender to enjoyment	−1.29	0.41	−3.11**	
	Enjoyment to practice	0.22	0.05	4.60**	
	Gender to practice (direct)	−0.23	0.23	1.00	
	Gender to practice (indirect)	−0.29	0.11		−2.59**

This was estimated using the indirect method (Preacher & Hayes, 2008)

* *p* < 0.05 and ** *p* < 0.01

#### An analysis of ratings of practice activities data previously collected in Study 1

At the end of the survey in Study 1 we had collected the same ratings of the practice activities included in that study like similar to the ratings made in Study 2B. A regression analysis predicting SCRABBLE skill from the subjective ratings found a significant effect of ratings of enjoyment, and ratings of enjoyment accounted for a significant amount of variance in SCRABBLE skill (*R*^*2*^ = 0.07, *p* < 0.05). Better players rated SCRABBLE-specific practice alone as more enjoyable *t* = 2.80, *p* < 0.01 and general word study as less enjoyable *t* = −2.42 *p* = 0.02. Ratings of enjoyment also discriminated males from females significantly, *χ*^2^ (3) = 15.21, *p* < 0.01. Again, females rated SCRABBLE-specific practice alone as less enjoyable p < 0.01 and rated playing SCRABBLE as more enjoyable *p* = 0.02.

We also tested whether gender differences in enjoyment of SCRABBLE-specific practice alone were related to differences in the other ratings of SCRABBLE-specific practice alone. Again, we found no direct effect of gender but a significant indirect effect through enjoyment, satisfying the criterion for full mediation. (See Table SOM4).

#### Individual differences in personality and gender

There were no significant differences in Grit and Harmonious Passion between the two genders (See Table 7). Only Obsessive Passion was significantly greater for males (*M* = 2.83, *SD* = 1.07) than for females (*M* = 2.39, *SD* = 1.24), *t*(120) = 2.13, *p* = 0.03). As a final check we tested if Obsessive Passion might predict the difference in enjoyment of SCRABBLE-specific practice alone. While in this model gender predicted passion *t*(120) = 2.13, *p* = 0.04) and enjoyment *t*(120) = −3.34, *p* < 0.01), Obsessive Passion did not have a direct effect on enjoyment *t*(120) = −1.08, *p* < 0.28. Therefore, the indirect relationship was not significant *z* < 1, with bootstrap confidence intervals showing a non-significant result.

### Discussion

Our goal with Study 2B was to search for variables that could explain the gender differences in engagement in effective practice activities, namely SCRABBLE-specific practice alone, which we previously established to be related to the acquisition of higher SCRABBLE skill. We followed the logic that variables that predicted gender and/or skill would be the best candidates to mediate the behavioral differences (which we had found in Study 2A) to be most pivotal in accounting for the gender differences in SCRABBLE skill.

We found that personality measures, such as Grit and Harmonious Passion did not differ between the genders and that Obsessive Passion was not a significant mediator of the association between gender and enjoyment in SCRABBLE-specific practice alone. Each of the personality variables significantly correlated with SCRABBLE ratings. Grit was significantly correlated with skill *r*(120) = 0.22, *p* = 0.02. Harmonious Passion was negatively related to skill *r*(120) = −0.20, *p* = 0.03. Obsessive Passion was positively related to skill *r*(120) = 0.20, *p* = 0.03. Finally, the personality variables did not significantly predict either skill or gender when other ratings, particularly the influential ratings of enjoyment, were statistically controlled.

Our analysis of the participants’ ratings of practice-related activities revealed surprising findings. The activity judged by the participants to be most relevant to improving one’s skill was playing SCRABBLE, which players rated much more relevant than SCRABBLE-specific practice alone. These ratings are inconsistent with the correlations in Study 1 and Study 2A, which found that amount of engagement in SCRABBLE-specific practice alone, but not SCRABBLE play, correlated with SCRABBLE skill. Our most important finding was that only the ratings of enjoyment of SCRABBLE practice activities predicted unique variance in gender and provided a full mediation of the observed significant relations between gender and amount of engagement in SCRABBLE activities, particularly SCRABBLE-specific practice alone.

The most important finding of Study 2B was that the rated subjective enjoyment of the different practice activities accounted for significant unique variance in gender and fully mediated the relation between gender and the engagement in SCRABBLE-specific practice alone. In sum, the ratings of enjoyment explain the gender difference in the behavior which best predicted SCRABBLE skill in Study 2A and was found to significantly mediate gender differences in skill in Study 1 and Study 2A.

## General discussion

Our studies supported the hypothesis that individual differences in skilled performance, at least in SCRABBLE, attributed to gender reflect gender differences in career history, defined by current practice, past practice, and prior experience. We found that individual differences in engagement in particular types of practice activities are correlated with gender, and when these effects of practice are statistically controlled, the gender-related effects were reduced (Study 1) and were no longer statistically significant (Study 2A). In Study 2B we found that gender difference in the involvement of different activities, particularly SCRABBLE-specific practice alone, was accounted for by perceived differences in enjoyment of those activities. While males and females did not significantly differ in their ratings of the relevance of these activities for improving skill or their difficulty, males reported finding them relatively more enjoyable, though neither group found the activities particularly enjoyable compared to other SCRABBLE-related activities. We believe that our findings are relevant to the study of individual differences in a large number of recreational and professional activities, where confirmed knowledge about practice activities that reliably improve performance is limited or completely lacking.

### Identification of practice activities that influence the development of SCRABBLE skill

For domains with a long tradition of training with professional instructors, there is a large body of research showing that a substantial proportion of individual differences in skilled performance is attributable to the differences in accumulated deliberate practice. Given that SCRABBLE does not have this body of developed knowledge about effective training, our study was forced to take an alternative and more inductive approach. Therefore, we collected information on the amount of engagement during their careers in a more diverse set of practice and play activities conducted alone or conducted with others. Our analyses in Study 1 showed that the amount of current engagement in SCRABBLE-specific practice alone was statistically significantly related to SCRABBLE skill, but the amount of time spent playing SCRABBLE was not. Our Study 2A showed that accumulated amount of SCRABBLE-specific practice alone was the best predictor of SCRABBLE ratings. The activities of SCRABBLE-specific practice alone match the practice activities in chess that have been found to be predictive of chess skill (Charness et al., 2005).

Our studies provide strong converging evidence for a basic model of skill acquisition in SCRABBLE, emphasizing SCRABBLE-specific practice activities alone and deemphasizing activities designed to build general vocabulary knowledge. This demonstrates that various practice variables had very different relationships with SCRABBLE skill ranging from positive (*β* = 0.46), to non-significant, to negative (*β* = −0.45) after controlling for the other practice variables. There have been recent attempts to propose a much simplified definition of deliberate practice as any structured domain activity (Macnamara, Hambrick, & Oswald, 2014). In contrast, our study suggests that purposeful practice cannot be measured by just any operationalization of practice hours, but that effective practice is much more specific and directly related to training activities that focus on changing aspects of the target performance (Ericsson, 2016; Ericsson et al., 1993). Some activities appear to be acting as suppressor variables with negative regression weights, which is inconsistent with the practice of measuring purposeful and deliberate practice by summing up all experience in the domain as proposed by Macnamara et al. (2014).

Study 2A offered an innovative approach for identifying practice-related activities where the amounts of engagement predicted attained skill. We collected data on many of these activities in SCRABBLE and conducted a factor analysis to identify different factors of co-varying levels of engagement in practice activities across the participants’ career in the domain. Our factor analysis identified five different types of practice activity factors, and our structural equation model showed that only the amount of engagement in SCRABBLE-specific practice alone positively related to SCRABBLE skill. In contrast, general vocabulary study was negatively predictive of attained SCRABBLE skill. Study 2A also identified factors associated with playing word games, non-tournament SCRABBLE play, and playing in SCRABBLE tournaments. Engagement in any of these three playing activities did not significantly correlate with attained SCRABBLE skill. Future research is needed to confirm if these factors are consistently identified and if they have a stable relation or lack of relation with skill across the full range of skill.

A theoretical analysis of appropriate, purposeful practice begins with an analysis of the target performance, namely superior performance during competition and tournaments. By generating actions in SCRABBLE situations for which superior actions are available, it is possible to identify cases where a given player does not find the best actions. Either by replaying published SCRABBLE games by elite players or consulting a SCRABBLE computer program, the player can get feedback on the quality of their move. If the selected move is not optimal, then the player needs to review how they generated their action and determine how they would have needed to proceed to generate the superior actions. This described activity corresponds well with the variable SCRABBLE-specific practice, for which we found that more engagement correlated with attained SCRABBLE skill.

In Study 2A we identified two factors related to practice one of which helped account for gender differences in tournament SCRABBLE. Females more frequently played SCRABBLE outside of tournaments than males and engaged less in SCRABBLE-specific practice alone. These differences in time allocation were sufficient to reduce and mediate the superior performance of males.

There are studies of individual differences in component skills and abilities that might provide insights into the acquisition and structure of superior performance in SCRABBLE. Tuffiash et al. (2007) measured different basic verbal abilities and verbal skills and showed that the only ability that distinguished experts from non-experts was solving anagrams (rearranging letters into words)—a task that experts reported practicing by themselves for a significantly longer duration than non-experts. Recently, Hargreaves, Pexman, Zdrazilova, and Sargious (2011) found that the superior verbal fluency of expert SCRABBLE players was only observed for tasks related to SCRABBLE.

### How can differences in engagement in different types of practice activities be explained

There are several different approaches to account for individual differences in engagement in practice activities. One approach is to collect data on how different participants perceive their practice activities regarding their relevance for improvement, the amount of effort required, and the amount of inherent enjoyment of those activities. An alternative approach is to search for correlations with differences in general personality traits. We collected data on three personality measures: Grit, Harmonious Passion, and Obsessive Passion. Our analysis found that the variables which uniquely explained variance in SCRABBLE skill involved perceptions of enjoyment of two types of practice activities, including the key variable of SCRABBLE-specific practice alone. In fact, enjoyment of SCRABBLE-specific practice alone fully mediated the gender differences in that activity.

Pinker’s (2008) general argument that males put more obsessive focus into their careers and competitive domains might explain males higher involvement than females in the more effective learning activities. This argument is also consistent with significantly higher Obsessive Passion scores of males compared to females. One problem with an account based on competitiveness is that we are not sure how to objectively measure competitiveness. The studies mentioned in the introduction experimentally manipulated competitive vs non-competitive situations, but SCRABBLE rating is based only on competitive situations. The closest objective measure we can think of is how often one plays SCRABBLE, and it is interesting that in Study 1 females engaged in statistically significantly more current SCRABBLE play (Table SOM1) while in Study 2A, there were no differences in the amount of tournament play. The only statistically significant differences we found in terms of reaction to competitive situations were that males reported Tournaments being more effortful while females reported playing SCRABBLE was more enjoyable. This could reflect a different approach to competition/playing SCRABBLE that might warrant further investigation.

### Generalizations of our study’s findings to other fields

Differences between genders are observed in some domains with expert performance, but not others. One domain without consistent gender effects is music. The domain of music is a domain with a very long history of training with developed curricula and professional teachers who start supervising training at a very young age. Ericsson et al.’s (1993) study of violinists found that all three groups of students at a music academy engaged in supervised practice, meeting the criteria for deliberate practice. In that domain, all groups had started practice at young ages—around age seven. All groups rated the relevance of practice alone as the most beneficial activity for improving skill. There is a significant body of knowledge about how to best improve at chess as well, and relatively few professional teachers who start supervising practice at more advanced levels with older players. In studies of the acquisition of expert performance in chess, Charness et al. (2005) found a stronger relation between chess skill and the age at the beginning of serious play than at the age of starting in the domain. Additionally, they found generally non-significant, positive effects of tournament play across different samples and types of samples. Most importantly, Charness et al. (2005) found evidence for an independent effect of engagement in purposeful practice for chess skill, even after controlling for other types of practice activities. Gobet and Campitelli (2007) also found that the amount of group practice (defined as playing chess, working with a tutor, or studying with another person) was a strong predictor of attained skill. When tournament chess players rated the relevance of different practice activities, Charness et al. (1996) found the average rating for analysis of games alone to be high (5.9 on a seven-point scale), whereas relevance of playing chess games outside tournaments and against chess computers was low, 3.6 and 3.2, respectively. Even more interesting, Charness et al. (1996) found that the ratings for the relevance of analyzing games alone were significantly correlated with chess ratings positively, whereas the ratings for the relevance of playing games were significantly negatively related. The relevance ratings show that the more skilled chess players knew which activities were associated with improved performance. In the recently established domain of SCRABBLE, we found hardly any evidence that players were able to accurately judge the relevance of different practice activities for improving performance. The players even rated playing SCRABBLE games outside tournaments as being more relevant than SCRABBLE-specific practice alone.

Gender differences in the amount of deliberate and purposeful practice have also been found in research on runners, where more men are distance runners, and male runners train more than female runners (Deaner, 2013). For instance, Ceci and Williams (2010) argue that the most important cause of the difference in professional outcomes is different preferences by women. Our studies have presented significant evidence that differences in performance between females and males are related to differences in the amount of engagement in particular types of practice activities, and that those differences in engagement in practice activities are related to differences in rated enjoyment of those activities. With our current evidence, we can only speculate about the causes of the difference in rated enjoyment of deliberate and purposeful practice.

## Limitations

There are a few important limitations to our studies. The most significant limitation concerns the correlational nature of the study. We cannot make statements about correlations with variables that we did not measure, including tests of basic abilities and other practice activities. In spite of our efforts to describe practice activities in SCRABBLE in more detail than previous research, there are several weaknesses in our use of retrospective reports of practice. It would, therefore, be significant to start collecting longitudinal information about players’ training. In a longitudinal study it would be possible to collect detailed diaries of SCRABBLE-related practice and perhaps discover other types of effective training activities. One potential problem with longitudinal studies is that training activities in new domains, such as SCRABBLE, may change over time as the domain matures and prize money is increased. For example, it is possible that individual tutoring may become more common and the amount of tutoring may become an important predictor of individual differences in performance.

Our study did not have our participants take tests of basic ability and intelligence, which merits discussion. Both Study 1 and Study 2 involved concurrence with experimental research on a smaller subsample of SCRABBLE players. Tuffiash et al. (2007) reported the results of the intensive study of the subsample, which was collected concurrently with Study 1. The corresponding data from the subsample examined in Study 2 have not yet been published. The general findings reported by Tuffiash et al. (2007) were that abilities predictive of SCRABBLE performance were closely related to SCRABBLE performance. These findings have been supported by Hargreaves et al. (2011), and in a recent neurological study by van Hees et al. (2016) where activities that are directly related to SCRABBLE show large differences as a function SCRABBLE skill, but other abilities or activities show very few significant relationships.

It is reasonable to speculate if the gender difference in SCRABBLE skill could be explained by differences in basic abilities. A compelling account of these differences regarding basic abilities would need to show a strong correlation between these abilities and SCRABBLE skill, ideally even within each gender. There are very few cognitive differences between females and males (Hyde, 2005). A very recent meta-synthesis found the average absolute value of cognitive gender difference is *d* = 0.22 with the biggest difference concerned mental rotation with *d* = −0.57 (favoring males) (Zell, Krizan, & Teeter, 2015). It is noteworthy that the gender differences observed in both our studies (−0.74 and −0.69) were numerically larger than any cognitive ability identified in the meta-synthesis. Spatial skills such as mental rotation seem to be a leading candidate because it shows consistent large gender differences in the population. In addition, Halpern and Wai (2007) reported significant correlation with SCRABBLE performance in their study. It should be noted though that if we statistically control for gender in the paper-folding task based on correlations reported by Halpern and Wai (2007), paper folding is no longer significantly related to rating (*p* = 0.053). On the other hand, if we control for performance on the paper-folding task, the correlations between gender and SCRABBLE rating is still significant (*p* < 0.001). Duffy, Ericsson, and Baluch (2007) similarly found that height, reach and gender were correlated with performance on darts. However, when gender was statistically controlled, height and reach were not significantly related to dart performance, yet when controlling for height and reach the relation between gender and dart performance remained highly significant. Both of these findings are consistent with the hypothesis that the developmental history involving training differs between genders and is a more likely source of the performance differences than these gender differences in height or paper-folding performance. Only future research will be able to provide deeper insights concerning if and how individual differences in basic abilities and attributes between males and females influence the acquisition of SCRABBLE performance.

## Future directions

Our Study 2B found that both males and females are acting quite rationally given how they reported their perception of the relevance of different types of practice for improved performance in SCRABBLE. We found that enjoyment was the main driver of gender differences in practice behavior. Future research might include experimental interventions attempting to change the perceptions of relevance and examine if such changes will change ratings of enjoyment and most importantly their engagement in the beneficial practice activities and ultimately associated increases in their performance during SCRABBLE tournaments. A more ambitious goal would be to promote the accumulation of knowledge about how SCRABBLE skill can be effectively trained under the supervision of qualified teachers (c.f. deliberate practice, Ericsson et al., 1993; Ericsson & Pool, 2016).

Another interesting direction would be to study other domains of expertise, where valid knowledge has not yet been developed about how individualized training with teachers can lead to high levels of performance. It is possible that such an approach might be useful when researchers study the extended preparation of engineers and scientists in STEM fields. Any intervention that helps a person come closer to maximizing their potential must obviously be offered to males as well, and there is no guarantee that, for instance, informing people of the benefits of fully concentrated study with opportunities to feedback will increase the amount of purposeful practice by females more than males.

## Conclusion

Research on SCRABBLE may provide us with insights and hypotheses about causes of gender differences in professional domains where measurement of performance is based nearly exclusively on subjective evaluations by supervisors and managers. This paper shows that a gender gap can arise in a domain with very few barriers to entry and where the common expectation is that women should prosper, as Halpern et al. (2011) demonstrated for SCRABBLE. We believe that domains of reproducibly superior expertise that exhibit a gender gap offer unique opportunities to study with objective measures how preferences, personality and, in our opinion most importantly, behavioral differences in the engagement in effective practice activities can produce a gender gap. We are excited about the possibility to study if and how preferences for engaging in effective practice can be influenced experimentally and if experimental manipulation can lead to increased long-term engagement in particular types of effective practice activities, which we hypothesize would lead to associated improvements in performance.

We have reviewed evidence supporting the claim that females and males gain roughly equivalent benefits from domain activities in SCRABBLE and are roughly equivalently able to succeed in the domain. The domain of SCRABBLE is likely similar to other domains of expertise where experts acquire unique domain-specific representations and cognitive processes (Ericsson & Kintsch, 1995). At this time, we have not found evidence for gender differences in the basic ability to acquire those representations. Therefore, we have proposed how large gender differences can be attributed to differences in the methods of skill acquisition as opposed to the capacity for skill acquisition or the rates of skill acquisition. We found that those differences appear to be due to preferences for engaging in certain types of domain-related activities. Future research is required to understand how these preferences originate, and most importantly, how those preferences can be changed by interventions.

### Electronic supplementary material

Below is the link to the electronic supplementary material.

Supplementary material 1 (DOCX 125 kb)
