# Supplementary material for: Gender differences in SCRABBLE performance and associated engagement in purposeful practice activities
Source: Psychol Res. 2017 Aug 31;83(6):1147–67. doi: 10.1007/s00426-017-0905-3 (PMC6647249; doi:10.1007/s00426-017-0905-3)
Supplement: Supplementary file 1 — Supplementary material 1 (DOCX 125 kb) [file 426_2017_905_MOESM1_ESM.docx]

**Supplemental Online Materials for**

Gender Differences in SCRABBLE Performance and Associated Engagement in Purposeful Practice Activities

Jerad H. Moxley

University of Miami Miller School of Medicine

K. Anders Ericsson, & Michael Tuffiash

Florida State University

A few analyses were not fully described in the paper so two of them are reported here, namely SOM-A1 and SOM-A2. These analyses were reported in earlier versions of the manuscript, but were removed due to space considerations. Following the summarized analyses are a number of tables that referred to in the text of the published manuscript or the summarized analyses, namely Tables SOM1, SOM2, SOM3, SOM4, SOM5, SOM6, SOM7 and SOM8.

**SOM-A1: Analysis of the Effects of Engagement in Different Types of Concurrent SCRABBLE Activity on SCRABBLE Ratings**

The effects of variables measuring the duration of engagement in SCRABBLE-related activities were used to predict current SCRABBLE ratings. In particular we assessed our hypothesis that the amount of engagement in some types of activities are better predictors of current SCRABBLE ratings than others. In the first regression model we entered the two accumulated variables and one variable adding up the hours of three current activities, namely SCRABBLE-specific practice alone, SCRABBLE play and general vocabulary study. In the second model we replaced the single variable with the sum of all current activities with two variables. One of the two variables summing up SCRABBLE play and the other variable summing up activities conducted alone (both SCRABBLE-specific practice alone and general vocabulary study). In the third and final model the amount of time conducted alone was split into the amount of SCRABBLE-specific practice alone and the amount of general vocabulary study. All of the variables summing up durations of activities were log transformed, and thus could not be described as linear combinations of each other. Therefore, we compared these models using Akaike and Bayesian information criterions, which are considered appropriate for testing non-nested hypotheses (Singer & Willet, 2003).

**Results**

The results of three regression analyses predicting ratings of SCRABBLE skill are reported in Table SOM2. The first regression model (Model 1) found significant effects of both cumulative variables (serious play and serious study alone), but the sum of the duration of all current SCRABBLE activities during a typical week is not significant. In Model 2 the sum of current SCRABBLE activities is replaced by two variables corresponding to the sum of SCRABBLE play and the sum of practice conducted alone, respectively. There is still no independent significant prediction of SCRABBLE skill by either of the two concurrent measures of SCRABBLE activity (see Table SOM2). In Model 3, the second composite variable of SCRABBLE practice conducted alone is replaced by two different variables, namely amount of concurrent vocabulary study and another variable measuring the time of analysis of SCRABBLE games and the time studying anagrams. This resulted in a significant improvement in the prediction of SCRABBLE ratings (see Table SOM2). The summed weekly current duration of the analysis of SCRABBLE games and studying anagrams is a significant positive predictor of SCRABBLE skill, whereas the duration of vocabulary study is a negative predictor of SCRABBLE skill. Hence, in the sample of tournament SCRABBLE players, if someone spends more time studying vocabulary then their SCRABBLE rating is predicted to be lower than a player matched on other variables, who spends less time studying vocabulary. The decision to separate the amount of current practice into three variables fits the data better than using only one or two variables describing practice and is supported by decreases in the BIC statistic (Rafferty, 1995). From Model 2 to Model 3 the BIC index decreases by 14 units and from Model 1 to Model 3 the BIC index was reduced by 11 units.

**SOM-A2 Analysis of Effects of Age, Starting Age, Experience, and Practice on SCRABBLE Ratings**

Once the best model was identified to explain SCRABBLE skill based on experience and practice in SOM-A1, we examined the effects of other variables, such as age and starting ages, to predict SCRABBLE ratings in the first step of a hierarchical multiple regression analysis. In the second step we entered gender to assess if any effects of gender remained significant. In the third step we entered interactions between the key demographic variables of age and gender and the various practice variables in a stepwise fashion. Given the Type I error inflation associated with the step-wise procedure, we will not interpret significant interactions but will include them for future replications.

**Results**

In the first step, we entered the variables from the best-fitting model identified in the first analysis (see Table SOM2) and also entered the demographic variables, namely starting age, current age and age of first tournament. As shown in Table SOM3, these eight variables measuring age, experience, and career trajectory made a statistically significant contribution to an account of SCRABBLE skill ratings, and together they accounted for over 59% of the variance F(8,126) = 22.91 *p* < .001. Four of the seven variables made a significant independent prediction of SCRABBLE ratings. Cumulative serious play, and current SCRABBLE-specific practice alone made positive significant contributions. The age of first playing in SCRABBLE tournaments made a significant negative contribution, but this means simply that the younger the age that a player played in their first SCRABBLE tournament predicts attaining a higher SCRABBLE rating. Finally, the amount of vocabulary study made a significantly negative contribution, where more vocabulary study predicts attaining a lower SCRABBLE rating.

In the second step of the hierarchical regression analysis we entered the variable of gender. This step was associated with a significant increase in the explained amount of variance in SCRABBLE skill, ΔF(1,125) = 15.16, p < .001. Even after controlling for practice, experience-related practice, and demographic variables, there was a significant effect of gender, where females were predicted to have a significantly lower level of SCRABBLE skill than men. Calculating an adjusted *d* based on the regression beta (Xie & Shauman, 2003) shows the effect of gender is still substantial *d_adj_* = -.52. The entry of gender to the regression did not change the significance of any of the four significant predictors in the first step. In the third step, interactions between practice variables and either age or gender were entered, but none of them reached statistical significance.

**Tables**

Table SOM1

Summary of correlations for SCRABBLE Rating (NSA), age, gender and first tournament age in Study 1 (N = 133)


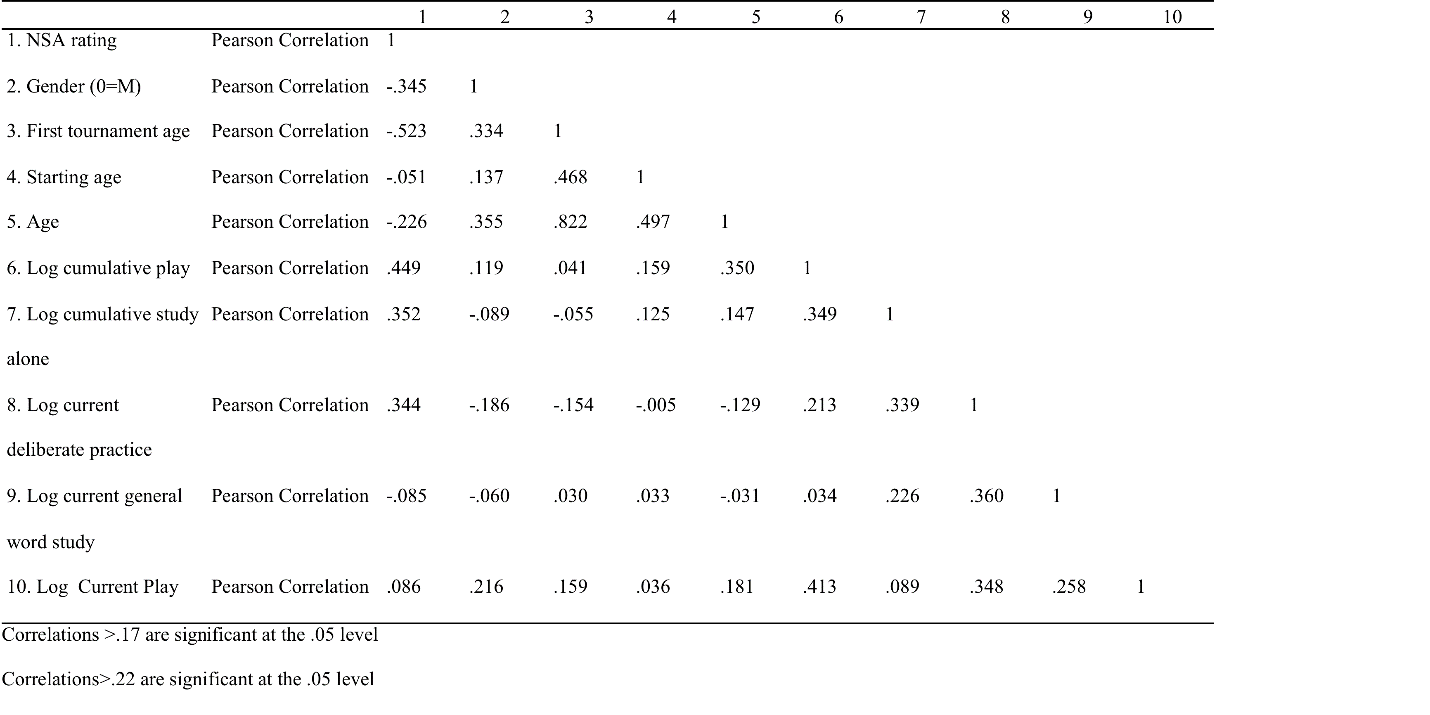


Table SOM2

Summary of relations between practice variables and SCRABBLE skill in Study 1. All variables are log transformed. (N = 135)

Model 1 Model 2 Model 3

Variable B SEM B SEM B SEM

Cumulative Serious Play 237.82** 50.31 259.51** 53.37 236.40** 50.01

Cumulative Study Alone 102.78** 35.59 85.64* 37.52 73.47* 35.24

Current Prac -77.61 86.64

Current SCRABBLE Play -133.19 88.84 -153.76 84.82

Current Study alone 63.99 80.15

Current Gen Word Study -221.51** 77.81

Current SCRABBLE study 346.37** 83.18

AIC 1935.74 1936.28 1919.50

BIC 1950.27 1953.71 1938.83

R^2^  .25 .26 .35

*Note *=p<.05 and **=p<.01*

Table SOM3

Summary of Hierarchical Multiple Regression for SCRABBLE Rating as Criterion for Study 1. All practice variables were log transformed. (N = 135)

Variable B SEM β f^2^ R^2^ ΔR^2^

Step 1 .59**

Starting age 4.78 2.51 .13 .03

First Tournament Age -18.40 3.20 -.69** .26

Cumulative Serious Play 191.16 47.68 .31** .13

Cumulative Study Alone 57.80 29.88 .13 .03

Current SCRABBLE

Specific Practice Alone 225.21 71.04 .22** .08

Current General Vocab -182.16 65.56 -.18** .06

Current SCRABBLE Play -11.24 71.89 .01 <.01.

Age 4.78 2.51 .13 .01

Step 2 .64** .04**

Gender -171.12 43.96 -.24** .12

*Note * = p < .05 and ** = p < .0*

Table SOM4

Summary of correlations for SCRABBLE Rating (NSA), age, gender and first tournament age in Study 2A (N = 122)


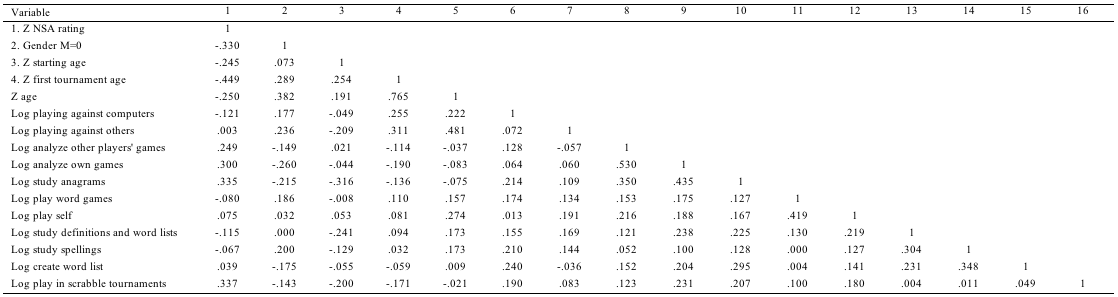


Correlations> .18 are significant at the .05 level

Correlations>.24 are significant at the .01 level

| Table SOM5  Standardized Item Loading Results for Structural Equation Model for Study 2 (N=122*)* | | | | |  |  |
| --- | --- | --- | --- | --- | --- | --- |
| Variable | SSPA | Splay | Games | Gen Voc | | Tournament |
| Play vs Others |  | .34** |  |  | |  |
| Play vs Computers |  | .22** |  |  | |  |
| Analyze Own Games | .78** |  |  |  | |  |
| Analyze Other Games | .62** |  |  |  | |  |
| Study Anagrams | .61** |  |  |  | |  |
| Play Word Games |  |  | .47** |  | |  |
| Play Self |  |  | .85** |  | |  |
| Study Definitions |  |  |  | .67** | |  |
| Study Spelling |  |  |  | .49** | |  |
| Create Word List |  |  |  | .41** | |  |
| Scrabble Tournaments |  |  |  |  | | 1.00** |

*Note*. **p* < .05. ***p* < .01. SSPA= SCRABBLE specific practice alone. Splay= Scrabble play, Gen Voc= General Vocabulary study.

Table SOM6

Summary of average ratings of various practice activities for relevance, effort, and enjoyment for Study 2B (n = 122).

Variable Relevance Effort Enjoyment

Competing in Tournaments Male 8.49 (2.10) 9.54 (.84)** 9.02 (1.32)

Competing in Tournaments Female 8.27 (2.28) 8.93 (1.58) 8.93 (1.66)

Competing in Tournaments All 8.38 (2.19) 9.23 (1.30) 8.97 (1.50)

Playing SCRABBLE Male 6.96 (2.00) 7.34 (1.59) 7.54 (1.71)

Playing SCRABBLE Female 7.29 (1.90) 7.44 (1.69) 8.29 (1.48)*

Playing SCRABBLE All 7.13 (1.95) 7.39 (1.64) 7.92 (1.63)

General Vocabulary Study Male 6.20 (2.56) 5.17 (2.52) 4.80 (2.53)

General Vocabulary Study Female 6.39 (2.46) 5.67 (2.34) 5.18 (2.38)

General Vocabulary Study All 6.30 (2.50) 5.43 (2.43) 5.00 (2.45)

Playing Games Male 4.01 (2.16) 6.18 (2.20) 6.34 (2.02)

Playing Games Female 3.94 (2.09) 5.84 (2.31) 6.12 (2.22)

Playing Games All 3.98 (2.12) 6.01 (2.26) 6.22 (2.12)

SCRABBLE specific practice alone Male 6.62 (2.59)* 5.95 (2.15)* 5.33 (2.09)**

SCRABBLE specific practice alone Female 5.54 (2.88) 4.98 (2.69) 4.04 (2.45)

SCRABBLE specific practice alone All 6.06 (2.78) 5.45 (2.48) 4.66 (2.37)

Note= *=t>1.98, p<.05, ** =t>2.61, p<.01. Star is placed in the column with the statistically significantly higher value.

TABLE SOM7

Summary of hierarchical multiple regression analysis of rating of various practice activities for relevance, effort, enjoyment, and personality measures entered as a set predicting SCRABBLE skill. And a multiple regression analysis with only sets that predict unique variance entered (N = 122).

Variable R^2^ Total R^2^ Unique B SEM t f^2^ R^2^

Relevance .13* .01

Joy .27** .12**

Effort .10* .02.

Personality .09* .03

Final Multiple Regression with enjoyment ratings 27

SCRABBLE Tournament

Play Joy .02 .02 76 <.01

Playing SCRABBLE Joy -.10 .03 -3.86 .13**

General vocabulary Joy -.04 .02 -2.51 .06*

Playing Games Joy .01 .02 .62 <.01

SCRABBLE specific practice

alone Joy .06 .02 3.37 .11**

*Note * = p < .05 and ** = p < .01*

Table SOM8

Mediation analysis testing if gender differences in subjective rating of activities mediates gender differences in actual participation in the activity for Study 1. This was estimated using the indirect method (Preacher & Hayes, 2008).

Practice Variable Path B SEM t-score Z-score

SCRABBLE specific practice alone

Gender to Enjoyment -.97 .39 -2.47*

Enjoyment to Practice .07 .01 5.72**

Gender to Practice (direct) -.07 .04 -1.48

Gender to Practice (indirect) -.07 .03 2.28*

*Note * = p < .05 and ** = p<.01*
